# Description of the first global outbreak of mpox: an analysis of global surveillance data

**DOI:** 10.1016/S2214-109X(23)00198-5

**Published:** 2023-06-20

**Authors:** Henry Laurenson-Schafer, Nikola Sklenovská, Ana Hoxha, Steven M Kerr, Patricia Ndumbi, Julia Fitzner, Maria Almiron, Luis Alves de Sousa, Sylvie Briand, Orlando Cenciarelli, Soledad Colombe, Meg Doherty, Ibrahima Soce Fall, Christian García-Calavaro, Joana M Haussig, Masaya Kato, Abdi Rahman Mahamud, Oliver W Morgan, Pierre Nabeth, Jeremias Domingos Naiene, Wildo Araujo Navegantes, Opeayo Ogundiran, Charles Okot, Richard Pebody, Tamano Matsui, Hugo López-Gatell Ramírez, Catherine Smallwood, Raúl Francisco Pérez Tasigchana, Aisling M Vaughan, George Sie Williams, WHO mpox Surveillance and Analytics team, Basma Abdelgawad, Amarnath Babu, Evans Buliva, Finlay Campbell, Daniel Cardoso Portela Câmara, Zainab Eleiba, Blanche Johanna Greene-Cramer, Esther Hamblion, Mahmoud Hassan, Kaja Kaasik-Aaslav, Basant Mohamed, Victoria Ndarukwa, James Richard Otieno, Jeffrey Pires, Jukka Pukkila, Felix Sanni, Craig Schultz, Tika Sedai, Laila Skrowny, Manilay Phengxay, Ariuntuya Ochirpurev, Jozica Skufca, Laura Goddard, Viema Biaukula, Peter Omondi Mala, Rosamund F Lewis, Boris I Pavlin, Olivier le Polain de Waroux

**Affiliations:** a Health Emergencies Programme, WHO, Geneva, Switzerland; b CPC Analytics, Berlin, Germany; c WHO Regional Office for the Americas, Washington, DC, USA; d European Centre for Disease Prevention and Control, Solna, Sweden; e WHO Regional Office for Europe, Copenhagen, Denmark; f Ministerio de Salud, Santiago, Chile; g WHO Regional Office for South-East Asia, Delhi, India; h WHO Regional Office for the Eastern Mediterranean, Cairo, Egypt; i WHO Regional Office for Africa, Brazzaville, Republic of the Congo; j WHO Regional Office for the Western Pacific, Manila, Philippines; k Secretaría de Salud, Mexico City, Mexico; l Ministerio de Salud Publica, Quito, Ecuador

## Abstract

**Background:**

In May 2022, several countries with no history of sustained community transmission of mpox (formerly known as monkeypox) notified WHO of new mpox cases. These cases were soon followed by a large-scale outbreak, which unfolded across the world, driven by local, in-country transmission within previously unaffected countries. On July 23, 2022, WHO declared the outbreak a Public Health Emergency of International Concern. Here, we aim to describe the main epidemiological features of this outbreak, the largest reported to date.

**Methods:**

In this analysis of global surveillance data we analysed data for all confirmed mpox cases reported by WHO Member States through the global surveillance system from Jan 1, 2022, to Jan 29, 2023. Data included daily aggregated numbers of mpox cases by country and a case reporting form (CRF) containing information on demographics, clinical presentation, epidemiological exposure factors, and laboratory testing. We used the data to (1) describe the key epidemiological and clinical features of cases; (2) analyse risk factors for hospitalisation (by multivariable mixed-effects binary logistic regression); and (3) retrospectively analyse transmission trends. Sequencing data from GISAID and GenBank were used to analyse monkeypox virus (MPXV) genetic diversity.

**Findings:**

Data from 82 807 cases with submitted CRFs were included in the analysis. Cases were primarily due to clade IIb MPXV (mainly lineage B.1, followed by lineage A.2). The outbreak was driven by transmission among males (73 560 [96·4%] of 76 293 cases) who self-identify as men who have sex with men (25 938 [86·9%] of 29 854 cases). The most common reported route of transmission was sexual contact (14 941 [68·7%] of 21 749). 3927 (7·3%) of 54 117 cases were hospitalised, with increased odds for those aged younger than 5 years (adjusted odds ratio 2·12 [95% CI 1·32–3·40], p=0·0020), aged 65 years and older (1·54 [1·05–2·25], p=0·026), female cases (1·61 [1·35–1·91], p<0·0001), and for cases who are immunosuppressed either due to being HIV positive and immunosuppressed (2·00 [1·68–2·37], p<0·0001), or other immunocompromising conditions (3·47 [1·84–6·54], p=0·0001).

**Interpretation:**

Continued global surveillance allowed WHO to monitor the epidemic, identify risk factors, and inform the public health response. The outbreak can be attributed to clade IIb MPXV spread by newly described modes of transmission.

**Funding:**

WHO Contingency Fund for Emergencies.

**Translations:**

For the French and Spanish translations of the abstract see Supplementary Materials section.

## Introduction

Mpox (formerly known as monkeypox), an infectious disease caused by the monkeypox virus (MPXV), was discovered in 1958 and first reported in humans in 1970. Before 2017, mpox primarily occurred in central and western Africa, with sustained MPXV transmission in local animal reservoirs and sporadic spillover into human populations, mostly in rural areas.^1^ Ongoing human-to-human transmission in western Africa has occurred since 2017 and led to occasional exportation to other countries from 2018 to 2021.^1^ The virus is primarily transmitted from person to person through direct contact with infected lesions and bodily fluids, but transmission through respiratory droplets and via contact with fomites might occur.^1^, ^2^ Two genetic clades of the MPXV exist—clade I and clade II, which includes subclades IIa and IIb.^3^

In May 2022, several countries with no historical sustained community transmission of mpox began reporting cases with no recent travel history to areas with known transmission and without links to imported animals (appendix 3 p 7). The first confirmed cases were in a family cluster and among sexual health services attendees in the UK, and cases rapidly increased and expanded to many countries, mainly among men who have sex with men (MSM), initially within Europe and subsequently to other regions. In July 2022, following consultation of the Emergency Committee convened under the International Health Regulations (IHR; 2005), the WHO Director-General declared the mpox multi-country outbreak to be a Public Health Emergency of International Concern.^4^ In line with the IHR, WHO issued temporary recommendations to support countries and guide a coordinated global response, including global mpox surveillance guidance.

**Research in context**

**Evidence before this study**

Since May, 2022, an unprecedented outbreak of mpox has been spreading in countries across the globe, including in areas with no previous zoonotic or community transmission of mpox. We conducted a literature search using the terms “monkeypox” and “variole du singe” and “mpox” in PubMed, Cochrane, MedRxiv, and bioRxiv for articles published in English and French from April 14, 2022, until January 31, 2023. In the context of our routine event-based surveillance activities, we also monitored publications from WHO, European Centre for Disease Prevention and Control, US Centers for Disease Control and Prevention, UK Health Security Agency, and other government public health websites, articles cited in the media, and Google searches for mpox studies. We also regularly consulted the mpox living evidence literature search and review updated regularly by the Public Health Agency of Canada.

Before 2022, available information on mpox pertained to cases in the African context, one outbreak in one country linked to importation of infected small mammals, and a few cases documented in relation to travel between 2018 and 2021. Since the beginning of the 2022–23 multi-country outbreak, several studies have been published describing the epidemiological and clinical characteristics of mpox cases. These studies were generally smaller in size and often limited to regional, national, or local contexts.

**Added value of this study**

This is the first global description of the 2022–23 multi-country mpox outbreak. This Article provides global context to previous and concurrent descriptions of the epidemiology of the outbreak, presents new and evolving features of this infectious disease, and highlights differences in regional trends. Our results corroborate other reports that, in this outbreak, the virus has spread predominantly through sexual contact among individuals identifying as men who have sex with men and has resulted in a range of modified or new clinical features compared with what had been observed in previous outbreaks. We also found that people who are very young (aged <5 years), older (aged >65 years), and immunosuppressed, have higher risk for hospitalisation due to mpox.

**Implications of all the available evidence**

Our study adds to the existing body of evidence indicating that mpox epidemics can be sustained through human-to-human transmission in a susceptible global population, including through sexual contact. These findings have important implications for mpox prevention and control, including case detection, contact tracing, prevention, risk communication, clinical care of patients, and research. Furthermore, our results can inform and guide public health policies and strategies designed to limit person-to-person transmission of monkeypox virus, improve equitable access to health care and vaccination, strengthen surveillance among communities at risk, encourage further investigation of the zoonotic origins and transmission of mpox, and support careful monitoring of changes in epidemiological profile in different contexts.

In this Article, we describe the global epidemiology of mpox cases reported to WHO by Member States through the global surveillance system.

## Methods

### Study design and participants

This is an analysis based on surveillance data reported to WHO by Member States (hereafter referred to as countries) under Article 6 of the IHR. Data were submitted by countries in a variety of formats and collated by WHO. Genomic sequencing data were obtained from the open-source databanks GISAID and GenBank, to which countries, institutions, and laboratories can upload genomic data. Therefore, no ethics approval or informed consent of participants was required to conduct the study.

### Procedures

WHO established a global surveillance system in May, 2022 (retrospectively expanded to Jan 1, 2022, and onwards) featuring two components: (1) daily aggregated numbers of mpox cases by country, and (2) a case reporting form (CRF) containing a minimum set of variables for each confirmed and probable mpox case,^5^ based on agreed upon case definitions (appendix 3 p 1).^6^ The CRF contains information on demographics, medical history, clinical presentation, epidemiological exposure factors, and laboratory testing.^5^ CRFs from the WHO European Region were reported by countries to WHO and the European Centre for Disease Prevention and Control through The European Surveillance System. Some countries retrospectively updated CRFs when new information was made available.

Data on sex, defined as sex at birth, were reported by the clinician completing the CRF based on self-reporting of cases, and the options were: female, male, other, and unknown. Gender was given as an option for reporting but was poorly completed and results are not shown.

All mpox cases reported to WHO were included in the analysis.

### Outcomes

The main outcome of the study was a descriptive analysis of the epidemic by time, person, and place and characterisation of trends. In addition to reporting cases, hospitalisation, and death by geographical region and country, we analysed symptom presentation by demographic group, and explored risk factors for hospitalisation for a range of demographic and other variables.

### Statistical analysis

We limited our analysis to confirmed mpox cases reported between Jan 1, 2022, and Jan 29, 2023. Analysis was performed for a range of sociodemographic, clinical, and epidemiological variables. All analyses were performed in R (version 4.2.1).

We used multivariable mixed-effects binary logistic regression to explore the risk factors for hospitalisation for age, sex, and four combinations of HIV and immunosuppressed status, incorporating country as a random effect. Cases that were hospitalised for isolation purposes were not considered hospitalised for this analysis. Immunosuppression as reported in the CRF included any immunosuppressive medication or disease as determined by the clinician and was considered separately from HIV status in this analysis. Regression was performed via the lme4 package in R.^7^ Further details of model fitting are described in appendix 3 (pp 1–2).

We estimated the effective reproduction number (R_eff_) over time, and modelled date of infection for confirmed cases using the EpiNow2 package.^8^ We assumed a mean incubation period of 8·1 days (SD 1·8) with a log-normal distribution^9^, ^10^ and a mean generation interval of 12·5 days (SD 9·6) with a gamma distribution.^11^ The reporting delay distribution, defined as the difference in days from date of symptom onset to date of notification at the national level, assumes a log-normal distribution with a maximum delay of 40 days. The truncation distribution, defined as the difference in days from the date of notification at the national level to the date WHO was notified, assuming a log-normal distribution with a maximum delay of 14 days. Our approach assumed that surveillance, testing, case definition, and data truncation were consistent over time and that all cases had been locally transmitted. Only countries with a reporting completeness above 70% (the proportion of total confirmed cases for which CRFs were submitted) and a minimum of 100 CRFs were included. Further details are provided in appendix 3 (p 2).

For the phylogenetic analysis, we de-duplicated sequences observed in both GISAID and GenBank (via SeqKit).^12^ Sequences were removed if they met one or more of the following criteria: sequences under 32 kbp in length; more than 1% of nucleotide residues were unknown (N); or sequences were not of good quality after Nextclade^13^ assessment. Phylogenetic analysis was performed with ParSNP^14^ using the monkeypox virus genomic sequence from an imported case from Nigeria in the UK in 2018 (MT903344.1)^15^ as a reference.

### Role of the funding source

The funder of the study had no role in study design, data collection, data analysis, data interpretation, or writing of the report.

## Results

**Figure 1:**
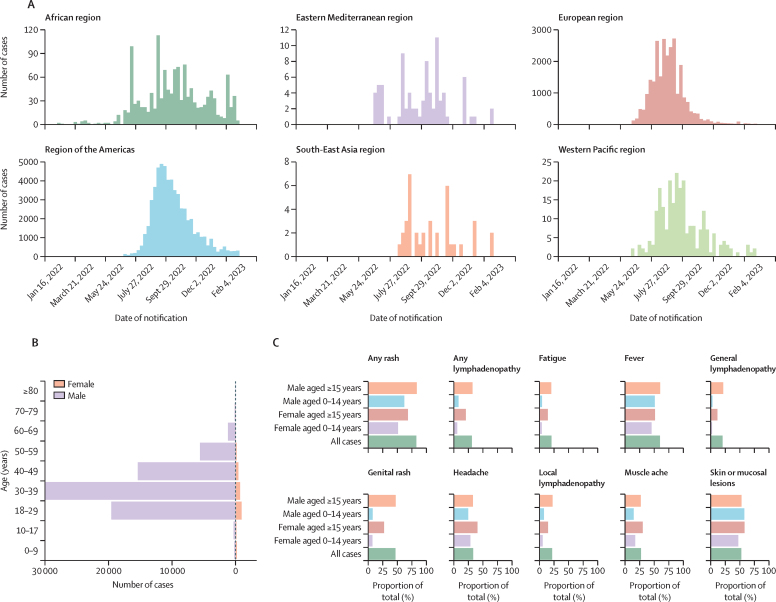
Mpox confirmed cases reported globally and their main characteristics, from Jan 1, 2022, to Jan 29, 2023 (A) Epidemic curves in all WHO regions. Note different y-axis scales for each region. The European region and the region of the Americas reported by far the highest number of cases with declining case counts at the end of this reporting period. The African region shows an unclear trend due to inconsistent reporting frequencies and more diverse patterns of transmission; other regions had largely sporadic cases and clusters. (B) Age and sex distribution of all cases. Cases were majority male and were most common among those aged between 18 and 50 years. (C) Frequency of symptoms among population groups stratified by sex and age. Of note, frequency of genital rash varied widely between population groups. Note that the skin or mucosal lesions exclude oral and genital lesions.

Between Jan 1, 2022, and Jan 29, 2023, 85 473 confirmed cases of mpox, including 89 deaths, were reported to WHO from 110 countries in all six WHO Regions (figure 1A). CRFs were submitted for 82 807 (96·9%) of all cases reported, with variance in coverage and completeness between WHO Regions and their constituent countries (appendix 3 p 4, 8).

Among cases with available demographic information, 73 560 (96·4%) of 76 293 were male, with a median age of 34 years (IQR 29–41). 25 938 (86·9%) of 29 854 men self-identified as MSM including gay, bisexual, and other MSM (table 1).

**Table 1:** Demographic and clinical characteristics of mpox cases where data were available, by age and WHO region each case was reported from

	**Sex**				**Sexual orientation**			**Health worker**	**Hospitalised**	**Admitted to intensive care unit**	**Total**
	Male	Female	Other	Unknown	Men who have sex with men	Other	Unknown				
**African region**											
0–4 years	28 (11·4%)	36 (23·1%)	0	0	NA	NA	NA	0	3 (5·4%)	0	64 (16·0%)
5–14 years	50 (20·4%)	36 (231%)	0	0	0	0	16 (6·0%)	0	8 (14·%)	0	86 (21·4%)
15–44 years	151 (61·6%)	74 (47·4%)	0	0	0	0	225 (84·3%)	0	38 (67·9%)	0	225 (56·1%)
45–64 years	14 (5·7%)	8 (5·1%)	0	0	0	0	22 (8·2%)	0	7 (12·5%)	0	22 (5·5%)
≥65 years	2 (0·8%)	0	0	0	0	0	2 (0·7%)	0	0	0	2 (0·5%)
Unknown	0	2 (1·3%)	0	0	0	0	2 (0·7%)	0	0	0	2 (0·5%)
Total	245	156	0	0	0	0	267	0	56	0	401
**Region of the Americas**											
0–4 years	97 (0·2%)	76 (3·6%)	0	6 (0·1%)	NA	NA	NA	0	16 (0·6%)	0	179 (0·3%)
5–14 years	141 (0·3%)	107 (5·1%)	0	2 (<0·1%)	3 (<0·1%)	45 (0·7%)	23 (0·1%)	0	9 (0·3%)	0	250 (0·4%)
15–44 years	40 793 (84·8%)	1560 (74·0%)	2 (100%)	3172 (49·4%)	12 783 (90·5%)	5768 (85·8%)	26 976 (76·1%)	956 (86·1%)	2226 (82·7%)	25 (67·6%)	45 527 (80·4%)
45–64 years	6765 (14·1%)	316 (15·0%)	0	701 (10·9%)	1310 (9·3%)	835 (12·4%)	5637 (15·9%)	151 (13·6%)	414 (15·4%)	8 (21·6%)	7782 (13·7%)
≥65 years	243 (0·5%)	44 (2·1%)	0	34 (0·5%)	25 (0·2%)	74 (1·1%)	222 (0·6%)	2 (0·2%)	24 (0·9%)	4 (10·8%)	321 (0·6%)
Unknown	72 (0·1%)	6 (0·3%)	0	2501 (39·0%)	3 (<0·1%)	2 (<0·1%)	2574 (7·3%)	1 (0·1%)	4 (0·1%)	0	2579 (4·6%)
Total	48 111	2109	2	6416	14 124	6724	35 432	1110	2693	37	56 638
**Eastern Mediterranean region**											
0–4 years	0	0	0	0	NA	NA	NA	0	0	0	0
5–14 years	0	1 (25·0%)	0	0	0	0	0	0	0	0	1 (1·8%)
15–44 years	48 (90·6%)	3 (75·0%)	0	0	17 (94·4%)	33 (89·2%)	1 (100%)	1 (100%)	4 (100%)	0	51 (89·5%)
45–64 years	4 (7·5%)	0	0	0	1 (5·6%)	3 (8·1%)	0	0	0	0	4 (7·0%)
≥65 years	0	0	0	0	0	0	0	0	0	0	0
Unknown	1 (1·9%)	0	0	0	0	1 (2·7%)	0	0	0	0	1 (1·8%)
Total	53	4	0	0	18	37	1	1	4	0	57
**European region**											
0–4 years	13 (0·1%)	10 (2·3%)	0	0	NA	NA	NA	0	2 (0·3%)	0	23 (10·5%)
5–14 years	7 (<0·1%)	3 (0·7%)	0	0	0	0	4 (<0·1%)	0	0	0	10 (<0·1%)
15–44 years	19 369 (77·3%)	348 (81·1%)	10 (83·3%)	34 (68·0%)	8177 (77·2%)	2587 (78·6%)	8997 (77·4%)	94 (87·9%)	486 (82·1%)	5 (71·4%)	19 761 (77·4%)
45–64 years	5408 (21·6%)	60 (14·0%)	2 (16·7%)	7 (14·0%)	2326 (22.0%)	662 (20·1%)	2489 (21·4%)	13 (12·1%)	99 (16·7%)	1 (14·3%)	5477 (21·4%)
≥65 years	233 (0·9%)	8 (1·9%)	0	0	79 (0·8%)	36 (1·1%)	126 (1·1%)	0	5 (0·8%)	1 (14·3%)	241 (0·9%)
Unknown	21 (0·1%)	0	0	9 (18·0%)	8 (0·1%)	8 (0·2%)	14 (0·1%)	0	0	0	30 (0·1%)
Total	25 051	429	12	50	10 590	3293	11 630	107	592	7	25 542
**South-East Asia region**											
0–4 years	0	0	0	0	NA	NA	NA	0	0	0	0
5–14 years	0	0	0	0	0	0	0	0	0	0	0
15–44 years	20 (90·9%)	16 (100%)	0	0	3 (75·0%)	19 (95·0%)	14 (100%)	0	31 (93·9%)	0	36 (94·7%)
45–64 years	2 (9·1%)	0	0	0	1 (25·0%)	1 (5·0%)	0	0	2 (6·1%)	0	2 (5·3%)
≥65 years	0	0	0	0	0	0	0	0	0	0	0
Unknown	22	16	0	0	4	20	14	0	33	0	38
**Western Pacific region**											
0–4 years	0	0	0	0	NA	NA	NA	0	0	0	0
5–14 years	0	0	0	0	0	0	0	0	0	0	0
15–44 years	50 (64·1%)	5 (100%)	0	0	3 (100%)	24 (25·3%)	28 (84·8%)	2 (66·7%)	9 (90·0%)	0	55 (42·0%)
45–64 years	7 (9·0%)	0	0	0	0	2 (2·1%)	5 (15·2%)	1 (33·3%)	1 (10·0%)	0	7 (5·3%)
≥65 years	0	0	0	0	0	0	0	0	0	0	0
Unknown	21 (26·9%)	0	0	48 (100%)	0	69 (72·6%)	0	0	0	0	69 (52·7%)
Total	78	5	0	48	3	95	33	3	10	0	131
**All regions**											
0–4 years	138 (0·2%)	122 (4·5%)	0	6 (0·1%)	NA	NA	NA	0	21 (0·6%)	0	266 (0·3%)
5–14 years	198 (0·3%)	147 (5·4%)	0	2 (<0·1%)	3 (<0·1%)	45 (0·4%)	43 (0·1%)	0	17 (0·5%)	0	347 (0·4%)
15–44 years	60 431 (82·2%)	2006 (73·8%)	12 (85·7%)	3206 (49·2%)	20 983 (84·8%)	8431 (82·9%)	36 241 (76·5%)	1053 (86·2%)	2794 (82·5%)	30 (68·2%)	65 655 (79·3%)
45–64 years	12 200 (16·6%)	384 (14·1%)	2 (14·3%)	708 (10·9%)	3638 (14·7%)	1503 (14·8%)	8153 (17·2%)	165 (13·5%)	523 (15·4%)	9 (20·5%)	13 294 (16·1%)
≥65 years	478 (0·6%)	52 (1·9%)	0	34 (0·5%)	104 (0·4%)	110 (1·1%)	350 (0·7%)	2 (0·2%)	29 (0·9%)	5 (11·4%)	564 (0·7%)
Unknown	115 (0·2%)	8 (0·3%)	0	2558 (39·3%)	11 (<0·1%)	80 (0·8%)	2590 (5·5%)	1 (0·1%)	4 (0·1%)	0	2681 (3·2%)
Total	73 560	2719	14	6514	24 739	10 169	47 377	1221	3388	44	82 807

Data are n (%) or n. Columns are not mutually-exclusive. Note that health worker status is distinct from those that acquired infection in health-care settings. NA=not applicable.

Of all cases reported with available data, only 903 (1·1%) of 80 126 were children aged 0–17 years (of which 266 were aged <5 years, and 347 of which were aged 5–15 years). The proportion of children varied between WHO regions, with a higher proportion of children in the African region (162 [40·6%] of 399). Lower proportions were seen in the Eastern Mediterranean region (two [3·6%] of 56), the region of the Americas (652 [1·2%] of 54 059), and the European region (87 [0·3%] of 25 512). No cases in children were reported in the South-East Asia region or the Western Pacific region. 57 women were reported as pregnant and one woman was reported as less than 6 weeks postpartum among the 2006 female cases aged 15–44 years (56 from the region of the Americas and two from the European region).

Health workers represented 1221 (5·3%) of 28 549 cases where health worker status was reported. Where information on mode of transmission was available, 32 (10·1%) of 316 reported occupational exposure: 27 while providing health care to patients and five in a clinical laboratory. The remaining health workers reported having been infected through non-occupational exposure, largely via sexual exposure.

Among 35 329 cases with known HIV status, 16 961 (48·0%) were HIV-positive. The proportion of people living with HIV was higher in the region of the Americas (12 997 [52·4%] of 24 816) than in the European region (3950 [37·8%] of 10 440). Fewer than 50 cases with known HIV status were reported in each of the other regions.

The most common symptoms reported were any rash (30 225 [82·6%] of 36 609 cases with reported symptoms), fever (21 819 [59·6%] of 36 609), skin or mucosal lesions (excluding oral and genital lesions; 19 121 [52·2%] of 36 609), genital rash (16 993 [46·4%] of 36 609), and headache (12 016 [32·8%] of 36 609). Symptom prevalence varied by age and sex (figure 1C; appendix 3 p 6). Notably, prevalence of genital rash was lower in female cases aged 15 years and older with reported symptoms (329 [27·0%] of 1218) than male cases aged 15 years and older with reported symptoms (16 201 [47·1%] of 34 362). This prevalence was also lower for female (11 [7·0%] 157) and male cases (14 [7·3%] of 191) younger than 15 years. 438 cases with reported genital rash symptoms did not have age and sex data reported. Prevalence of genital rash varied among regions, with the lowest rates in the Eastern Mediterranean region (18 [32·1%] of 56) and highest rates in the Western Pacific region (35 [80·0%] of 44). Symptom data were extremely limited for the African region.

The most reported mode of transmission was via sexual encounter (14 941 [68·7%] of 21 749; table 2). Reported modes of transmission varied between regions, with sexual transmission comprising 9133 (94·0%) of 9711 likely transmission events from the European Region, 14 (93·3%) of 15 likely events from the South-East Asian region, eight (80%) of ten likely events from the Western Pacific region, 5780 (48·2%) of 12 000 likely events from the region of the Americas, and six (46%) of 13 likely events from the Eastern Mediterranean region. Reported transmission settings varied: the most common setting was in small gatherings with sexual contact such as a night club, private party, or sauna (3470 [66·3%] of 5223 settings). Other reported settings were the household (553 [10·6%] of 5223 settings) and large events (such as festivals or sport events) without sexual contact (236 [4·5%] of 5223 settings; appendix 3 p 5).

**Table 2:** Transmission mode of mpox cases where data were available, by case demographic characteristics

	**Contact with contaminated material (n=303)**	**Health-care associated (n=94)**	**Vertical transmission during pregnancy or birth (n=1)**	**Person-to-person (n=2374)**	**Sexual encounter (n=14 941)**	**Animal to human (n=13)**	Occupational exposure*(n=9)	**Parenteral transmission**†**(n=2)**	**Other (n=4012)**	**Total (n=21 749)**
**Age group**										
0–9 years	9 (19·6%)	1 (2·2%)	1 (2·2%)	15 (32·6%)	1 (2·2%)	0	0	0	19 (41·3%)	46
10–17 years	5 (5·9%)	2 (2·4%)	0	13 (15·3%)	31 (36·5%)	0	0	0	34 (40·0%)	85
18–29 years	97 (1·6%)	32 (0·5%)	0	611 (10·0%)	3859 (63·2%)	6 (0·1%)	3 (<0·1%)	1 (<0·1%)	1497 (24·5%)	6106
30–39 years	122 (1·4%)	30 (0·3%)	0	1027 (11·4%)	6183 (68·6%)	6 (0·1%)	3 (<0·1%)	1 (<0·1%)	1641 (18·2%)	9013
40–49 years	49 (1·0%)	22 (0·5%)	0	519 (10·9%)	3452 (72·8%)	1 (<0·1%)	1 (<0·1%)	0	697 (14·7%)	4741
50–59 years	17 (1·2%)	3 (0·2%)	0	155 (11·0%)	1130 (80·2%)	0	1 (0·1%)	0	103 (7·3%)	1409
60–69 years	2 (0·7%)	1 (0·3%)	0	21 (7·1%)	255 (85·9%)	0	1 (0·3%)	0	17 (5·7%)	297
70–79 years	1 (2·6%)	2 (5·1%)	0	9 (23·1%)	23 (59·0%)	0	0	0	4 (10·3%)	39
≥80 years	1 (16·7%)	1 (16·7%)	0	1 (16·7%)	3 (50·0%)	0	0	0	0	6
Unknown	0	0	0	3 (42·9%)	4 (57·1%)	0	0	0	0	7
**Sex**										
Female	39 (6·7%)	30 (5·2%)	1 (0·2%)	94 (16·2%)	232 (39·9%)	0	4 (0·7%)	0	182 (31·3%)	582
Male	262 (1·2%)	64 (0·3%)	0	2278 (10·8%)	14 691 (69·5%)	13 (0·1%)	5 (<0·1%)	2 (<0·1%)	3830 (18·1%)	21 145
Other	1 (8·3%)	0	0	1 (8·3%)	10 (83·3%)	0	0	0	0	12
Unknown	1 (10·0%)	0	0	1 (10·0%)	8 (80·0%)	0	0	0	0	10
**Sexual orientation**										
Men who have sex with men	142 (1·0%)	27 (0·2%)	0	1294 (9·4%)	10 252 (74·4%)	3 (<0·1%)	0	0	2055 (14·9%)	13 773
Other	111 (3·3%)	43 (1·3%)	0	264 (7·9%)	1192 (35·5%)	10 (0·3%)	6 (0·2%)	0	1729 (51·5%)	3355
Unknown	50 (1·1%)	24 (0·5%)	1 (<0·1%)	816 (17·7%)	3497 (74·7%)	0	3 (0·1%)	2 (<0·1%)	228 (4·9%)	4621

Data are n (%) unless otherwise stated. Note that demographic groups are not mutually exclusive. When reporting cases, country officials have the ability to report more than one mode of transmission. The denominators used for the percentage calculations are the totals in the far right column. The most common form of transmission was sexual encounter, with markedly higher rates in men, men who have sex with men, and individuals aged 18–49 years.

* Laboratory.

† Transfusion or intravenous drug use.

Within the African region, demographic characteristics varied between west Africa and central Africa (appendix 3 p 9). CRFs were available for 401 (30·8%) of 1302 cases from nine countries in total within western Africa (Benin, Ghana, Liberia, and Nigeria), central Africa (Cameroon, Central African Republic, Congo [Brazzaville], and the Democratic Republic of the Congo), and southern Africa (South Africa). In western Africa, there was a higher percentage of male cases than female cases (133 [68·5%] of 194 *vs* 61 [31·4%] of 194); overall median age of 29 years (IQR 18–37). In contrast, in central Africa, cases were younger than those reported globally or in west Africa (median age 14 years [IQR 6–29]), and had an even sex distribution (107 [53%] of 202 were male and 95 [47·0%] were female). In both west and central Africa, where cases were reported before 2022, there was a substantial increase in the number of confirmed cases in 2022 relative to previous years—in particular, Nigeria shows a steady increase of cases during the reporting period.

Of the 54 117 cases globally for which information was available, 3927 (7·3%) were hospitalised. Of those, 1112 (28·3%) were for treatment, 539 (13·7%) were for isolation, and 2276 (58·0%) were for unknown reasons. 44 patients were reported as admitted to the intensive care unit (ICU).

For our risk factor analysis, the 539 cases hospitalised for isolation were not considered hospitalised. Here, when compared with those aged 15–44 years, both cases aged 65 years or older (adjusted odds ratio [aOR] 1·54 [95% CI 1·05–2·25], p=0·026) and children aged 0–4 years had significantly increased odds to be hospitalised (aOR 2·12 [1·32–3·40], p=0·0020 table 3). Additionally, when compared with cases who were HIV-negative and immunocompetent, cases who were both HIV-positive and immunocompromised were found to have increased odds of hospitalisation (aOR 2·00 [1·68–2·37], p<0·0001), as were cases who were immunocompromised and HIV-negative (aOR 3·47 [1·84–6·54], p=0·0001; table 3). Having a reported positive HIV status alone did not increase the odds of hospitalisation (aOR 0·91 [0·71–1·16], p=0·44). However, cases with both unknown HIV and immunocompetence status had significantly increased odds to be hospitalised (aOR 1·16 [1·03–1·31], p=0·014). Finally, female cases had higher odds of hospitalisation than male cases (aOR 1·61 [1·35–1·91], p<0·0001).

**Table 3:** Risk factors for hospitalisation among confirmed mpox cases

	**Frequencies**		**Unadjusted OR**		**Adjusted OR**	
	Not admitted (n=50 525)	Hospitalised (n=3388)	OR (95% CI)	p value	OR (95% CI)	p value
**Age group**						
15–44 years	41 657 (93·7%)	2794 (6·3%)	NA	NA	NA	NA
0–4 years	149 (88·0%)	21 (12·0%)	2·10 (1·29–3·25)	0·0015	2·12 (1·32–3·40)	0·0020
5–14 years	214 (92·6%)	17 (7·4%)	1·18 (0·70–1·88)	0·50	0·81 (0·44–1·51)	0·51
45–64 years	8163 (94·0%)	523 (6·0%)	0·96 (0·87–1·05)	0·35	0·97 (0·88–1·08)	0·60
≥65 years	315 (91·6%)	29 (8·4%)	1·37 (0·92–1·97)	0·10	1·54 (1·05–2·25)	0·026
Unknown	27 (87·0%)	4 (13·0%)	2·21 (0·65–5·65)	0·14	1·98 (0·76–5·16)	0·16
**Sex**						
Male	46 906 (93·9%)	3053 (6·1%)	NA	NA	NA	NA
Female	1816 (90·7%)	187 (9·3%)	1·58 (1·35–1·84)	<0·0001	1·61 (1·35–1·91)	<0·0001
Unknown	1803 (92·4%)	148 (7·6%)	1·26 (1·06–1·49)	0·0080	1·35 (1·13–1·62)	0·0013
**HIV and immunocompromised status**						
HIV- and not immunosuppressed	8445 (94·9%)	457 (5·1%)	NA	NA	NA	NA
HIV+ and not immunosuppressed	1540 (94·9%)	82 (5·1%)	0·98 (0·77–1·25)	0·90	0·91 (0·71–1·16)	0·44
HIV- and immunosuppressed	73 (86·9%)	11 (13·1%)	2·78 (1·39–5·06)	0·0017	3·47 (1·84–6·54)	0·0001
HIV+ and immunosuppressed	4387 (93·9%)	287 (6·1%)	1·21 (1·04–1·41)	0·014	2·00 (1·68–2·37)	<0·0001
Unknown	36 080 (93·4%)	2551 (6·6%)	1·31 (1·18–1·45)	<0·0001	1·16 (1·03–1·31)	0·014

Data are n (%) unless otherwise stated. Table includes unadjusted ORs, and results of multivariable binomial regression, where country was included as a random effect (not shown). Multivariable model included age, sex, and HIV and immunocompromised status as a predictor variables. The denominators used for calculating the frequency percentages is the sum of the number of cases in the not admitted and hospitalised columns. Older age groups (aged ≥65 years), children (aged <5 years), and immunocompromised individuals, regardless of HIV status, were most at risk for hospitalisation. NA=not applicable. OR=odds ratio.

Proportionally, more pregnant women were hospitalised than non-pregnant women aged 15–44 years (12 [26·1%] of 46 *vs* 137 [9·2%] of 1493).

In the reporting period, 89 mpox-related deaths (crude case fatality ratio 0·1%) were reported to WHO (67 from the region of the Americas, 15 from the African region, five from the European region, one from the Eastern Mediterranean region, and one from the South-East Asian region), and case-based information was available for 30 (33·7%) of the 89 deaths. Of these 30 all were male, and 21 (84·0%) of 25 for whom sexual orientation was reported had self-identified as MSM. The median age was 30 years (IQR 27·5–40·0) and 23 (85·2%) of 27 for whom HIV status was available reported living with HIV. Almost all cases, where data were available, 22 (91·6%) of 24 were immunosuppressed, 26 (86·6%) of 30 had been admitted to hospital (20 for clinical need, five for unknown reasons, and one for isolation), and 12 (48·0%) of 25 had been admitted to ICU.

**Figure 2:**
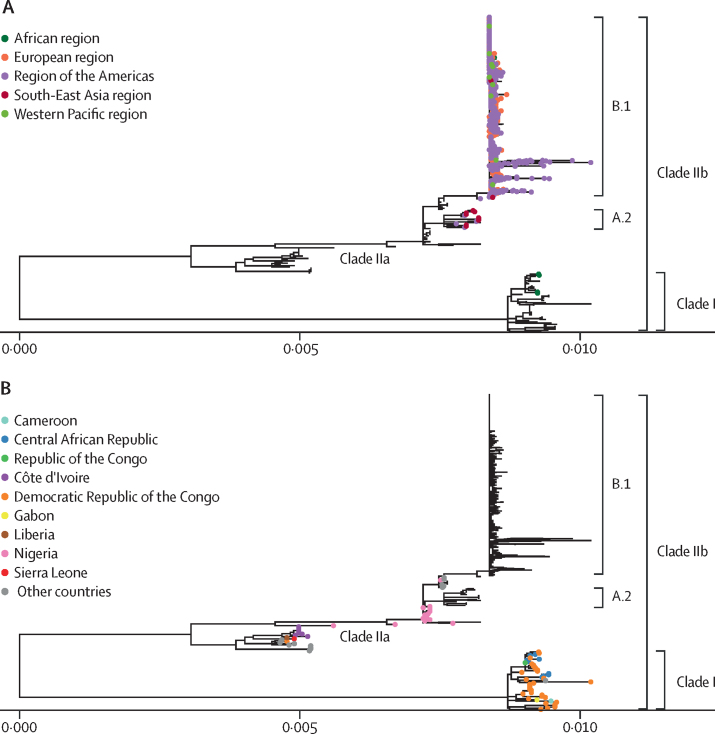
Phylogenetic trees of available monkeypox virus genomic sequences in public databases to Sept 20, 2022 (A) Genomic sequences from Jan 1, 2022, until Sept 20, 2022, highlighted with coloured tips according to WHO region. Outside of central Africa, all sequences are associated with clade IIb, with the majority falling under lineage B.1. (B) Genomic sequences sampled before 2022 shown with coloured tips by country, highlighting differences between cases in clade I and II. Countries described under other countries are located outside of the African continent, which had sporadic mpox cases before 2022.

As of Sept 20, 2022, a total of 1619 MPXV genomic sequences were analysed, with a large variation between countries in the proportion of cases with available sequencing data. All documented sequences with a collection date in 2022 belong to MPXV clade IIb, mainly lineage B.1 followed by lineage A.2, except for four clade I sequences from the Democratic Republic of the Congo (figure 2A). The 2022 outbreak sequences belonging to Clade IIb clustered with the 2018 and 2019 cases in the UK which were linked to travel from Nigeria (figure 2B).

**Figure 3:**
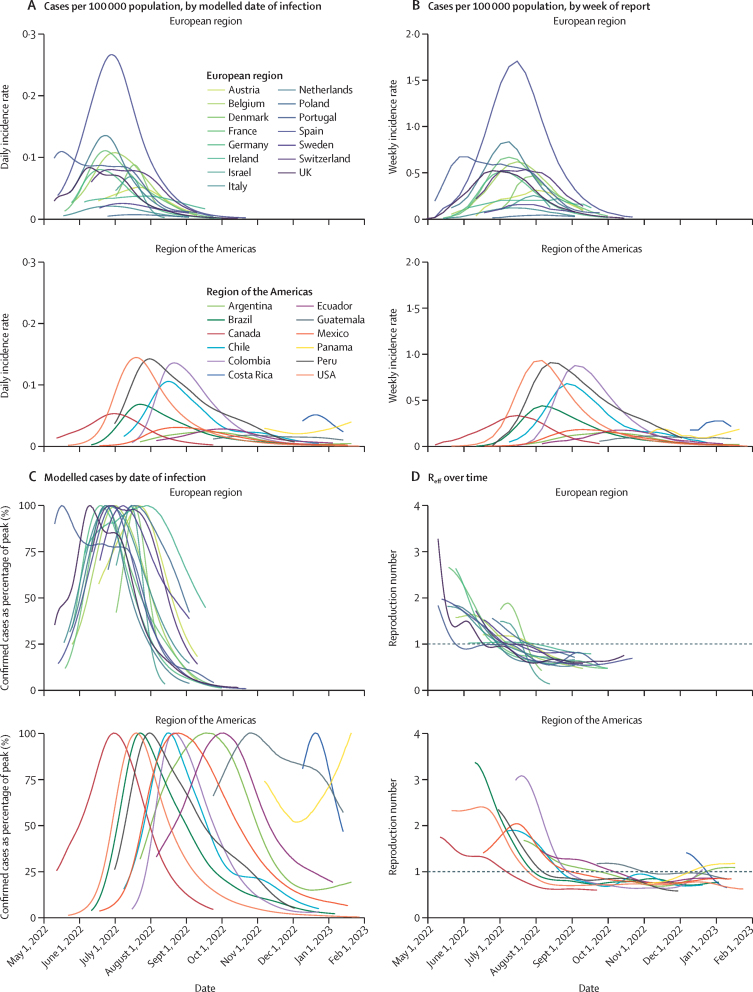
Trends in confirmed cases over time for the two WHO regions where modelling inclusion criteria were met, with each line representing a single country Weeks in which countries reported less than ten confirmed cases have been excluded due to uncertainty surrounding estimates based on low case numbers. (A) Daily incidence rate for countries shown as number of confirmed cases per 100 000 population, by modelled date of infection, split by region to show regional differences in trends. (B) Weekly incidence rates for countries shown as the number of cases per 100 000 population, shown by week of report. Incidence was estimated using population estimates from the UN Population Division. (C) Confirmed cases over time, by modelled date of infection, shown as a percentage of the peak number of infections. (D) Median estimated R_eff_ over time for countries which met the inclusion criteria. R_eff_=effective reproduction number.

Estimates for case trajectories by date of infection and R_eff_ were produced for 27 countries in Europe and the Americas. The early stage of the epidemic was driven by transmission in the European Region, where countries saw relatively homogeneous trajectories (appendix 3 p 10; figure 3). For 15 European countries for which the epidemic trajectory was modelled (Austria, Belgium, Denmark, France, Germany, Ireland, Israel, Italy, the Netherlands, Poland, Portugal, Spain, Sweden, Switzerland, and the UK), we estimated a peak in infections around the end of June (median June 28, 2022 [IQR June 23–July 16, 2022]) and a median peak in reported cases, by date of report, on July 16, 2022 (IQR July 9–July 30, 2022). R_eff_ for most countries in Europe fell below 1·0 in July, 2022 (median July 9, 2022 [IQR June 30–July 22, 2022]), with the 15 European countries observing R_eff_ of less than 1·0 by Aug 1, 2022. As of Jan 31, 2023, R_eff_ estimates remain below 1·0 for the European region, although relatively low case counts contribute to uncertainty surrounding these estimates.

The mpox epidemic in the region of the Americas lagged behind the European region by several weeks. Trends in the region were more heterogeneous, with one country, Panama, still observing rising weekly case counts at the end of January, 2023, with a 60% increase compared with December, 2022 (40 *vs* 25; appendix 3 p 11; figure 3). Additionally, as of Jan 29, 2023, the modelled growth rate was positive for Panama (1·4%), Argentina (0·7%), and Colombia (0·3%). For 12 countries for which the trajectory was modelled (Argentina, Brazil, Canada, Chile, Colombia, Costa Rica, Ecuador, Guatemala, Mexico, Panama, Peru, and the USA), our data suggest a median peak in new infections on Aug 23, 2022 (IQR July 29–Oct 9, 2022) and a median peak in reported cases, by date of report, on Sept 6, 2022 (IQR Aug 11–Oct 22, 2022). As of January, 2023, most countries in the region of the Americas observed relatively low case counts relative to their peaks, with the exception of Panama, Costa Rica, Paraguay, Guatemala, and El Salvador. From September to November, 2022, cases generally declined in the European and Americas regions. In the months following, community transmission continued at relatively low levels, with new clusters being reported regularly.

## Discussion

The 2022–23 epidemic is the largest-ever documented outbreak of mpox in terms of magnitude and geographical spread. Our results provide insights into its epidemiology and highlight features not previously documented. These insights include differences in regional trends, risk factors, demographics, and clinical characteristics among regions and settings.

This mpox outbreak, related to clade IIb MPXV and reported in mostly newly affected countries to have spread mainly among MSM, has been sustained by human-to-human transmission through sexual contact in various settings.

Many reported cases show a different clinical presentation than previously documented; the clinical presentations are less severe, a smaller proportion of the cases report symptoms during the prodromal phase, the eruptive phase has been marked by more anogenital and oropharyngeal lesions, and there has been less frequent generalised rash than previously reported.^16^, ^17^ Differences in skin lesion morphology probably stem from differences in routes and intensity of exposure^18^—supported by our findings indicating greater frequency of genital rash in men aged 18 years and older. More research is needed to better understand whether the often different clinical presentation than documented in the past is linked to heightened surveillance and better detection, lower viral load, mode of transmission and site of virus entry, specific characteristics of the current outbreak and clade, mild disease going undetected in Africa, or a combination of these factors.^17^, ^19^

Although detailed information on cases from the African region is limited, data on age, sex, place, and time of reporting suggest more heterogeneous transmission patterns. In the Central African Republic and the Democratic Republic of the Congo, where only clade I MPXV has been reported, cases are mainly children and young adults, almost evenly distributed by sex, clustered geographically in more rural areas,^20^ and without a clear epidemic pattern.^16^, ^21^ Similarly, an outbreak which began in August, 2022, linked primarily to clade I MPXV within Sudanese camps for internally displaced people and refugees, involved cases mostly in young children (data not shown). The observed difference in epidemiology between clade I and IIb cases might be attributable to a range of factors: including surveillance biases, viral, and host factors specific to areas where transmission occurs. In contrast, Nigeria has seen a rapid upsurge of cases since April, 2022, driven largely by clade IIb as confirmed by available sequence data, with an epidemiology and clinical features that more closely resemble those observed in newly affected countries. Here, cases are concentrated in urban areas and more prevalent among young males than females, and proportionally more children and women are affected than in the global outbreak.^22^ Cases of mpox in both women and men who report exposure through sex suggests that heterosexual transmission^23^ occurs as well as transmission between men.^24^ More data are needed to understand the epidemiological situation in the African context, the nature of human-to-human spread, as well as the fundamental role of zoonotic transmission.

Our analysis highlights increased odds of hospitalisation for children younger than 5 years, adults aged 65 and older, females, and individuals immunocompromised^25^ due to uncontrolled HIV or other immunocompromising conditions or treatments. Independent clinical studies have also found that uncontrolled HIV infection is a risk factor for severe disease,^19^, ^26^, ^27^, ^28^ while the higher odds of hospitalisation among older individuals and female cases had not been previously described. This finding might be due to smaller sample sizes and differing demographic age groups of previous mpox disease cohorts. Observed increased odds of hospitalisation in female cases might be due to health seeking behaviour, surveillance biases, transmission modality, or intrinsic host factors. These findings need to guide clinical management, and ensure adequate care for the immunocompromised, children, and older adults.

Finally, our findings reveal remarkable similarities in transmission dynamics across countries in the European region, with a uniform negative growth rate (decline in weekly case counts) reached by August, 2022.^29^ Transmission dynamics were more heterogeneous in the region of the Americas, reflecting the larger geographical scope and social, economic, and health-care system diversity of the region. Although assessing the effectiveness of public health interventions is beyond the scope of this Article, it is notable that declining case counts were observed in some countries before the introduction of vaccination, suggesting that other factors, such as infection induced immunity and behaviour changes in groups at risk, might have contributed to limiting transmission.^30^

This global analysis of the outbreak epidemiology was possible thanks to the sharing of case-based data with WHO. Nevertheless, case-based information was incomplete or not available for some countries and regions. Case-based data were not always updated so complications, hospitalisation, and ICU admission, which occurred after notification, might be under-reported and disease severity underestimated. Additionally, the focus of the outbreak among MSM might have introduced surveillance bias in identifying and testing for MPXV. Our analysis of phylogenetic data aimed to give an overview of the clades and lineages circulating in each region and further studies on virus evolution are needed to better interpret the observed variability.

Like most surveillance systems, our data probably underestimate the real number of mpox infections, and fear of stigma related to sexual orientation, especially in places where same-sex relationships are not socially accepted or are punishable by law, might have accentuated this underestimation and introduced reporting biases in some settings.

While the global outbreak is brought under control, efforts must continue to support mpox elimination and control efforts in all countries. Necessary actions include strengthening surveillance; enhancing diagnostic and genetic sequencing capacity, particularly in Africa; ensuring optimal health care and integrated sexual health and HIV prevention and care services, supporting appropriate immunisation services and advancing equitable access to vaccines and therapeutics; conducting risk communication to enhance ability to mitigate individual risk and to reduce stigma and fear; and supporting a One Health approach for outbreak investigation and research to minimise zoonotic transmission of MPXV. This event offers an opportunity to strengthen sub-national, national, regional, and global capacities and collaboration as well as invest in research and development to respond to emerging infectious disease threats.

## Data sharing

The aggregated and case-based data was shared with WHO by Member states under Article 6 of the International Health Regulation (2005). The data dictionary is defined in the Case Reporting Form, and the surveillance definitions and set-up are defined in the WHO Interim Guidance for surveillance, case investigation and contact tracing for mpox, which can be found at https://www.who.int/publications/i/item/WHO-MPX-Surveillance-2022.4. As defined in the Guidance, WHO uses case-based surveillance data only for its own products, including external peer review publications, to better understand and explain the epidemiology of the mpox outbreak for the benefit of all countries. Data cannot be shared with external third parties.

## Declaration of interests

We declare no competing interest.

## Supplementary Materials

French translation of the abstract

Spanish translation of the abstract

## References

[1] Gessain A, Nakoune E, Yazdanpanah Y (2022). Monkeypox. N Engl J Med.

[2] Jezek Z, Fenner F, Melnick JL (1988). Human monkeypox.

[3] Ulaeto D, Agafonov A, Burchfield J (2023). New nomenclature for mpox (monkeypox) and monkeypox virus clades. Lancet Infect Dis.

[4] WHO (July 23, 2022). WHO Director-General's statement at the press conference following IHR Emergency Committee regarding the multi-country outbreak of monkeypox - 23 July 2022. https://www.who.int/director-general/speeches/detail/who-director-general-s-statement-on-the-press-conference-following-IHR-emergency-committee-regarding-the-multi--country-outbreak-of-monkeypox--23-july-2022.

[5] WHO (Jan 23, 2023). Mpox (monkeypox) case investigation form (CIF) and minimum dataset case reporting form (CRF). https://www.who.int/publications/m/item/monkeypox-minimum-dataset-case-reporting-form-(crf).

[6] WHO (Dec 22, 2022). Surveillance, case investigation and contact tracing for monkeypox: interim guidance. https://www.who.int/publications/i/item/WHO-MPX-Surveillance-2022.4.

[7] Bates D, Mächler M, Bolker BM, Walker SC (2015). Fitting linear mixed-effects models using lme4. J Stat Softw.

[8] Abbott S, Hellewell J, Thompson RN (2020). Estimating the time-varying reproduction number of SARS-CoV-2 using national and subnational case counts. Wellcome Open Res.

[9] Miura F, van Ewijk CE, Backer JA (2022). Estimated incubation period for monkeypox cases confirmed in the Netherlands, May 2022. Euro Surveill.

[10] Charniga K, Masters NB, Slayton RB (2022). Estimating the incubation period of monkeypox virus during the 2022 multi-national outbreak. medRxiv.

[11] Guzzetta G, Mammone A, Ferraro F (2022). Early estimates of monkeypox incubation period, generation time, and reproduction number, Italy, May–June 2022. Emerg Infect Dis.

[12] Shen W, Le S, Li Y, Hu F (2016). SeqKit: A cross-platform and ultrafast toolkit for FASTA/Q file manipulation. PLoS One.

[13] Aksamentov I, Roemer C, Hodcroft E, Neher R (2021). Nextclade: clade assignment, mutation calling and quality control for viral genomes. J Open Source Softw.

[14] Treangen TJ, Ondov BD, Koren S, Phillippy AM (2014). The Harvest suite for rapid core-genome alignment and visualization of thousands of intraspecific microbial genomes. Genome Biol.

[15] Mauldin MR, McCollum AM, Nakazawa YJ (2022). Exportation of monkeypox virus from the African continent. J Infect Dis.

[16] Jezek Z, Grab B, Szczeniowski M, Paluku KM, Mutombo M (1988). Clinico-epidemiological features of monkeypox patients with an animal or human source of infection. Bull World Health Organ.

[17] Huhn GD, Bauer AM, Yorita K (2005). Clinical characteristics of human monkeypox, and risk factors for severe disease. Clin Infect Dis.

[18] Reynolds MG, Yorita KL, Kuehnert MJ (2006). Clinical manifestations of human monkeypox influenced by route of infection. J Infect Dis.

[19] Jezek Z, Szczeniowski M, Paluku KM, Mutombo M (1987). Human monkeypox: clinical features of 282 patients. J Infect Dis.

[20] Rimoin AW, Mulembakani PM, Johnston SC (2010). Major increase in human monkeypox incidence 30 years after smallpox vaccination campaigns cease in the Democratic Republic of Congo. Proc Natl Acad Sci USA.

[21] Kalthan E, Tenguere J, Ndjapou SG (2018). Investigation of an outbreak of monkeypox in an area occupied by armed groups, Central African Republic. Med Mal Infect.

[22] Nigeria Centre for Disease Control and Prevention An update of monkeypox outbreak in Nigeria. https://ncdc.gov.ng/diseases/sitreps/?cat=8&name=An%20Update%20of%20Monkeypox%20Outbreak%20in%20Nigeria.

[23] Ogoina D, James IH (2022). Monkeypox among linked heterosexual casual partners in Bayelsa, Nigeria. Qeios.

[24] Ogoina D, Izibewule JH, Ogunleye A (2019). The 2017 human monkeypox outbreak in Nigeria—report of outbreak experience and response in the Niger Delta University Teaching Hospital, Bayelsa State, Nigeria. PLoS One.

[25] Curran KG, Eberly K, Russell OO (2022). HIV and sexually transmitted infections among persons with monkeypox - eight U.S. jurisdictions, May 17–July 22, 2022. MMWR Morb Mortal Wkly Rep.

[26] O'Shea J, Filardo TD, Morris SB, Weiser J, Petersen B, Brooks JT (2022). Interim guidance for prevention and treatment of monkeypox in persons with HIV Infection — United States, August 2022. MMWR Morb Mortal Wkly Rep.

[27] Yinka-Ogunleye A, Aruna O, Dalhat M (2019). Outbreak of human monkeypox in Nigeria in 2017–18: a clinical and epidemiological report. Lancet Infect Dis.

[28] Ogoina D, Iroezindu M, James HI (2020). Clinical course and outcome of human monkeypox in Nigeria. Clin Infect Dis.

[29] Vaughan AM, Cenciarelli O, Colombe S (2022). A large multi-country outbreak of monkeypox across 41 countries in the WHO European Region, 7 March to 23 August 2022. Euro Surveill.

[30] Delaney KP, Sanchez T, Hannah M (2022). Strategies adopted by gay, bisexual, and other men who have sex with men to prevent monkeypox virus transmission — United States, August 2022. MMWR Morb Mortal Wkly Rep.

