# Supplementary material for: Description of the first global outbreak of mpox: an analysis of global surveillance data
Source: Lancet Glob Health. 2023 Jun 20;11(7):e1012–23. doi: 10.1016/S2214-109X(23)00198-5 (PMC10281644; doi:10.1016/S2214-109X(23)00198-5)
Supplement: French translation of the abstract [file mmc1.pdf]

THE LANCET
Global Health

Supplementary appendix 1

This translation in French was submitted by the authors and we reproduce it as supplied. It has not been peer reviewed. *The Lancet*'s editorial processes have only been applied to the original in English, which should serve as reference for this manuscript.

Cette traduction en français a été proposée par les auteurs et nous l'avons reproduite telle quelle. Elle n'a pas été examinée par des pairs. Les processus éditoriaux du *Lancet* n'ont été appliqués qu'à l'original en anglais et c'est cette version qui doit servir de référence pour ce manuscrit.

Supplement to: Laurenson-Schafer H, Sklenovská N, Hoxha A, et al. Description of the first global outbreak of mpox: an analysis of global surveillance data. *Lancet Glob Health* 2023; **11:** e1012–23.

Description de la première épidémie mondiale de mpox : une analyse des données de surveillance mondiale

Contexte : En mai 2022, plusieurs pays sans antécédents de transmission communautaire soutenue du mpox (anciennement connu sous le nom de la variole du singe) ont notifié à l'OMS de nouveaux cas de mpox. Ces cas ont rapidement été suivis par une épidémie à grande échelle qui s'est déployée dans le monde entier, entraînée par une transmission locale à l'intérieur des pays précédemment non touchés. Le 23 juillet 2022, l'OMS a déclaré l'épidémie comme une Urgence de Santé Publique de Portée Internationale. Cette étude a pour objectif de décrire les principales caractéristiques épidémiologiques de cette épidémie, la plus importante à ce jour.

Méthodes : Dans cette analyse des données mondiales de surveillance, nous avons analysé les données de tous les cas de mpox confirmés rapportés par les États membres de l'OMS via le système mondial de surveillance du 1er janvier 2022 au 29 janvier 2023. Les données comprenaient les chiffres quotidiens agrégés des cas de mpox par pays et un formulaire de déclaration de cas (FDC) contenant des informations sur les données démographiques, la présentation clinique, les facteurs d'exposition épidémiologiques et les tests de laboratoire. Nous avons utilisé les données pour (1) décrire les principales caractéristiques épidémiologiques et cliniques des cas; (2) analyser les facteurs de risque d'hospitalisation (par régression logistique binaire à effets mixtes multivariée); et (3) analyser rétrospectivement les tendances de transmission. Les données de séquençage provenant de GISAID et GenBank ont été utilisées pour analyser la diversité génétique du virus de la variole du singe (MPXV).

Résultats : Les données de 82 807 cas avec des FDC soumises ont été incluses dans l'analyse. Les cas étaient principalement dus à la clade IIb du MPXV (principalement la lignée B.1, suivie de la lignée A.2). L'épidémie était causée par une transmission entre hommes (73 560 [96·4%] des 76 293 cas) s'identifiant comme des hommes ayant des relations sexuelles avec des hommes (25 938 [86·9%] des 29 854 cas). La voie de transmission la plus couramment rapportée était le contact sexuel (14 941 [68,7%] des 21 749 cas). 3927 (7·3%) des 54 117 cas ont été hospitalisés, avec des chances accrues pour les moins de 5 ans (rapport de cotes ajusté 2,12 [IC à 95% 1·32-3·40], p=0,0020), les personnes âgées de 65 ans et plus (1,54 [1·05-2,25], p=0·026), les cas féminins (1·61 [1·35-1·91], p<0·0001), et les cas immunodéprimés soit en raison de leur statut VIH positif et d'une immunodépression (2·00 [1·68-2·37], p<0·0001), soit en raison d'autres conditions d'immunodépression (3·47 [1·84-6·54], p=0·0001).

Interprétation : La surveillance mondiale continue a permis à l'OMS de surveiller l'épidémie, d'identifier les facteurs de risque et d'informer la réponse de santé publique. L'épidémie peut être attribuée à la propagation de la clade IIb du MPXV par de nouveaux modes de transmission décrits récemment.

Financement : Fonds de contingence de l'OMS pour les urgences
