# Supplementary material for: Description of the first global outbreak of mpox: an analysis of global surveillance data
Source: Lancet Glob Health. 2023 Jun 20;11(7):e1012–23. doi: 10.1016/S2214-109X(23)00198-5 (PMC10281644; doi:10.1016/S2214-109X(23)00198-5)
Supplement: Spanish translation of the abstract [file mmc2.pdf]

THE LANCET
Global Health

Supplementary appendix 2

This translation in Spanish was submitted by the authors and we reproduce it as supplied. It has not been peer reviewed. *The Lancet*'s editorial processes have only been applied to the original in English, which should serve as reference for this manuscript.

Los autores nos proporcionaron esta traducción al español y la reproducimos tal como nos fue entregada. No la hemos revisado. Los procesos editoriales de *The Lancet* se han aplicado únicamente al original en inglés, que debe servir de referencia para este manuscrito.

Supplement to: Laurenson-Schafer H, Sklenovská N, Hoxha A, et al. Description of the first global outbreak of mpox: an analysis of global surveillance data. *Lancet Glob Health* 2023; **11:** e1012–23.

Descripción del primer brote global de mpox: un análisis de los datos de vigilancia global

Antecedentes: En mayo de 2022, varios países sin historial de transmisión comunitaria sostenida de mpox (anteriormente conocido como viruela del mono) notificaron a la OMS nuevos casos de mpox. Estos casos fueron seguidos rápidamente por un brote a gran escala que se extendió por todo el mundo, impulsado por la transmisión local dentro de países previamente no afectados. El 23 de julio de 2022, la OMS declaró el brote como una Emergencia de Salud Pública de Importancia Internacional. El objetivo de este estudio es describir las principales características epidemiológicas de este brote, el más grande reportado hasta la fecha.

Métodos: En este análisis de datos de vigilancia global, analizamos los datos de todos los casos confirmados de mpox reportados por los Estados Miembros de la OMS a través del sistema de vigilancia global desde el 1 de enero de 2022 hasta el 29 de enero de 2023. Los datos incluyeron números diarios agregados de casos de mpox por país y un formulario de notificación de casos (FNC) que contenía información sobre datos demográficos, presentación clínica, factores de exposición epidemiológica y pruebas de laboratorio. Utilizamos los datos para (1) describir las principales características epidemiológicas y clínicas de los casos; (2) analizar los factores de riesgo de hospitalización (mediante regresión logística binaria de efectos mixtos multivariable); y (3) analizar retrospectivamente las tendencias de transmisión. Se utilizaron datos de secuenciación de GISAID y GenBank para analizar la diversidad genética del virus de la viruela del mono (MPXV).

Resultados: Se incluyeron en el análisis los datos de 82,807 casos con FNC presentados. Los casos se debieron principalmente al clado IIb del MPXV (principalmente linaje B.1, seguido por linaje A.2). El brote fue impulsado por la transmisión entre hombres (73,560 [96·4%] de 76,293 casos) que se identifican a sí mismos como hombres que tienen relaciones sexuales con hombres (25,938 [86·9%] de 29,854 casos). La ruta de transmisión más comúnmente informada fue el contacto sexual (14,941 [68·7%] de 21,749 casos). Se hospitalizaron 3,927 (7·3%) de 54,117 casos, con mayores probabilidades para aquellos menores de 5 años (razón de momios ajustada 2·12 [IC del 95%: 1·32-3·40], p=0·0020), mayores de 65 años (1·54 [1·05-2·25], p=0·026), casos femeninos (1·61 [1·35-1·91], p<0·0001) y casos inmunosuprimidos debido a ser VIH positivos e inmunosuprimidos (2·00 [1·68-2·37], p<0·0001), o debido a otras condiciones de inmunocompromiso (3·47 [1·84-6·54], p=0·0001).

Interpretación: La vigilancia global continua permitió a la OMS monitorear la epidemia, identificar factores de riesgo e informar la respuesta de salud pública. El brote se puede atribuir a la propagación del MPXV del clado IIb mediante nuevos modos de transmisión descritos recientemente.

Financiamiento: Fondo de Contingencia para Emergencias de la OMS.
