# Supplementary material for: Description of the first global outbreak of mpox: an analysis of global surveillance data
Source: Lancet Glob Health. 2023 Jun 20;11(7):e1012–23. doi: 10.1016/S2214-109X(23)00198-5 (PMC10281644; doi:10.1016/S2214-109X(23)00198-5)
Supplement: Supplementary appendix 3 [file mmc3.pdf]

THE LANCET
Global Health

Supplementary appendix 3

This appendix formed part of the original submission and has been peer reviewed. We post it as supplied by the authors.

Supplement to: Laurenson-Schafer H, Sklenovská N, Hoxha A, et al. Description of the first global outbreak of mpox: an analysis of global surveillance data. *Lancet Glob Health* 2023; **11:** e1012–23.

Appendix

Detailed description of methods and supplementary findings

Mpox case and death definition

As per WHO guidance, the **case definition** for a confirmed mpox case is the following:

A person with laboratory confirmed MPXV infection by detection of unique sequences of viral DNA by real-time polymerase chain reaction (PCR) and/or sequencing.

It is also noted that: *PCR on a blood specimen may be unreliable and should also not be used alone as a first line diagnostic test. If blood PCR is negative and was the only test done, this is not sufficient to discard a case that otherwise meets the definition of a suspected for probable case. This applies regardless of whether the blood PCR was for Orthopoxvirus or MPXV specific.*

This definition has been consistent throughout WHO guidance released during the 2022 mpox outbreak. It should be noted that this definition may not be used by all WHO Member States, and that confirmed cases reported to WHO may adhere to national case definitions.

A **mpox death for surveillance purposes** is defined as a death in a probable or confirmed mpox case, unless the alternative cause of death is trauma. The diagnosis for mpox can also be confirmed after the death has occurred if there is sufficient lesion material to perform PCR testing.

Risk factor analysis

For risk factor analysis, hospitalisation was considered only for those that were hospitalised for treatment, or for unknown reasons. Those that were hospitalised for isolation purposes were considered not hospitalised. In our model, only countries where at least one case was hospitalised, and one case was not hospitalised were considered.

Due to high levels of missingness for the HIV/immunocompromised variable, we also analysed the characteristics of records with missing variables, or whether missing occurred at random. This variable was defined as the intersection of the HIV and immunocompetence variables. We looked at cases that were:

i. HIV- and not immunosuppressed
ii. HIV+ and not immunosuppressed
iii. HIV- and immunosuppressed
iv. HIV+ and immunosuppressed
v. Of unknown status

In defining our model, the Akaike information criterion (AIC) of the null binary fixed-effects model and the null binary mixed-effects models were compared, and a likelihood ratio test (LRT) was also performed. In both cases, the mixed-effects models had a lower AIC value, and via the LRT, increased the explanatory power of the model with $p < 0.05$. In addition, after testing for inclusion of mixed effects, the inclusion of age group, sex, and the combined HIV/immunocompetence variable in four categories outlined above as a fixed effect was tested for each via the same criteria. In both cases, inclusion of the fixed effect reduced AIC and significantly increased the explanatory power of the model via LRT. In addition, each model was checked for overdispersion via Pearsons Chi-square test.

The **results of model fitting** are shown here:

- The null model with no random effects had a higher AIC (25311) compared to the null model with country included as a random effect (22875). Via likelihood ratio test, inclusion of random effects significantly increased the explanatory power of the model ($p<0.0001$)
- Inclusion of age into the null model with random effects was consistent with lower AIC, (22872 vs 22875) and increased explanatory power via LRT ($p = 0.017$). The model was not significantly overdispersed via Pearson’s Chi-square ($p = 1.00$).
- Inclusion of HIV/immunocompetence into the model was consistent with lower AIC, (22814 vs 22872) and increased explanatory power via LRT ($p <0.0001$). The model was not significantly overdispersed via Pearson’s Chi-square ($p = 0.99$).
- Inclusion of sex into the model was consistent with lower AIC, (22784 vs 22814) and increased explanatory power via LRT ($p <0.0001$). The model was not significantly overdispersed via Pearson’s Chi-square ($p = 0.99$).

Data from the following countries were included in the model:

Argentina, Austria, Belgium, Brazil, Bulgaria, Canada, Central African Republic, Chile, China, Colombia, Costa Rica, Cuba, Cyprus, Czechia, Denmark, Dominican Republic, Ecuador, El Salvador, Estonia, Georgia, Greece, Guatemala, Guyana, Hungary, Iceland, India, Ireland, Italy, Jamaica, Japan, Latvia, Lebanon, Lithuania, Luxembourg, Malta, Netherlands, Nigeria, Norway, Panama, Paraguay, Peru, Poland, Portugal, Qatar, Republic of Korea, Republic of Moldova, Romania, Slovakia, Slovenia, Spain, Sri Lanka, Sudan, Sweden, Thailand, The United Kingdom, Ukraine, United States of America

R_{eff} projections

We estimated the effective reproduction number (R_{eff}) over time using the EpiNow2 package. We use a generative R_{eff} model to estimate latent infections then map these modelled cases by date of infection to observed cases via reporting delays. Each time series is fit independently using Markov-chain Monte Carlo (MCMC). This process uses four chains to estimate infections with 250 warmup samples and 2,000 post-warmup samples per chain to estimate median R_{eff} along with 20%, 50%, and 90% credible intervals.

We obtained our assumptions for mean incubation period and mean generation interval from estimates in the scientific literature obtained in the ongoing 2022-23 outbreak. The reports from which these estimates are obtained are cited in the main text and mentioned in the methods. Country-specific reporting delay distributions were obtained from variables in the case reporting form (CRF) (the time difference between date of onset and date of report to national authorities). Country-specific truncation

distributions were obtained via database statistics (the delay between cases being notified at national level to entry in WHO databases).

R_{eff} estimates are only shown for weeks in which ten or more cases were observed in a country to preserve quality of estimates. Only countries with a reporting completeness above 70% (the proportion of total confirmed reported cases for whom CRFs were submitted) and a minimum of 100 CRFs were included.

The following countries met our eligibility criteria: Argentina, Austria, Belgium, Brazil, Canada, Chile, Colombia, Costa Rica, Denmark, Ecuador, France, Germany, Guatemala, Ireland, Israel, Italy, Mexico, Netherlands, Panama, Peru, Poland, Portugal, Spain, Sweden, Switzerland, The United Kingdom, United States of America.

Supplementary tables

Table S1

Proportion of mpox cases with case reporting forms (CRF) submitted, by WHO region, 1 January 2022 to 29 January 2023

WHO Region	Confirmed cases (n)	Confirmed cases with case details (n)	Detailed cases reported (%)
Region of the Americas	57,971	56,638	97.7
European Region	25,804	25,542	99.0
African Region	1,343	401	29.9
Western Pacific Region	235	131	56
Eastern Mediterranean Region	82	57	70
South-East Asia Region	38	38	100

Table S1 Reporting completeness by WHO region, with the number of confirmed cases reported and submitted with details provided from the WHO case reporting form (CRF), as a proportion of all confirmed cases reported.

Table S2

Exposure settings of mpox cases reported to WHO, by key demographic characteristics, 1 January 2022 to 29 January 2023

	Household n (%)	School/nursery n (%)	Large event		Small event		Workplace n (%)	Healthcare n (%)	Other n (%)	Total n (%)
			No sexual contact n (%)	Sexual contact n (%)	No sexual contact n (%)	Sexual contact n (%)				
**Age group (years)**										
0-9	9 (90)	1 (10)	0	0	0	0	0	0	0	10
10-17	5 (33)	0	2 (13)	0	1 (7)	4 (27)	0	0	3 (20)	15
18-29	159 (13·4)	0	50 (4·2)	52 (4·4)	32 (2·7)	727 (61·4)	17 (1·4)	4 (0·3)	143 (12·1)	1,184
30-39	220 (10·6)	2 (0·1)	96 (4·6)	100 (4·8)	53 (2·6)	1,353 (65·3)	21 (1·0)	3 (0·1)	223 (10·8)	2,071
40-49	90 (6·8)	0	70 (5·3)	57 (4·3)	35 (2·6)	933 (70·1)	13 (1·0)	5 (0·4)	128 (9·6)	1,331
50-59	29 (6)	0	18 (4)	12 (2)	14 (3)	366 (75)	4 (1)	1 (0)	43 (9)	487
60-69	11 (10)	0	0	0	5 (5)	76 (70)	2 (2)	1 (1)	13 (12·0)	108
70-79	3 (21)	0	0	2 (14)	1 (7)	8 (57)	0	0	0	14
80+	0	0	0	0	0	1 (100)	0	0	0	1
Unknown	0	0	0	0	0	2 (100)	0	0	0	2
**Sex**										
Female	46 (42·2)	0	9 (8)	1 (1)	4 (4)	32 (29)	3 (3)	3 (3)	11 (10)	109
Male	478 (9·4)	3 (0·1)	227 (4)	222 (4·3)	137 (2·7)	3,437 (67·3)	54 (1·1)	11 (0·2)	540 (10·6)	5,109
Other	2 (40)	0	0	0	0	1 (20)	0	0	2 (40)	5
**Sexual orientation**										
MSM	390 (8·8)	1 (0·0)	157 (3·5)	198 (4·5)	90 (2·0)	3,102 (69·9)	30 (0·7)	4 (0·1)	463 (10·4)	4,435
Other	87 (19)	1 (0)	35 (8)	20 (4·3)	19 (4·10)	199 (43.0)	14 (3)	6 (1)	82 (18)	463
Unknown	39 (12)	0	44 (14)	5 (2)	32 (10)	169 (54)	13 (4)	4 (1)	8 (3)	314
**Total**										
	526	3	236	223	141	3,470	57	14	553	5,223

Table S2: Likely exposure setting of mpox cases where data were available, by case demographic characteristics. Note that demographic groups are not mutually exclusive. Table values are shown as the number of cases (n) and the percentage of the row. When reporting cases, country officials have the ability to report more than one likely exposure setting per case. The most common exposure setting was in small events with sexual contact. The large and small events were defined as such – small: sexual contact at night club/private party/sauna or similar setting vs Bar/restaurant/ or other small event where there was no sexual contact; large - Large event with sexual contact vs Large event with no sexual contact (e.g., festival or sports event).

Table S3

Symptom frequencies of mpox cases reported to WHO, by case age and sex, 1 January 2022 to 29 January 2023

Symptom	All cases (n = 36,609) n (%)	Male, 15+ (n = 34,362) n (%)	Female, 15+ (n = 1,218) n (%)	Male, 0-14 (n = 191) n (%)	Female, 0-14 (n = 157) n (%)
Any rash	30,225 (82·6)	28,600 (83·2)	831 (68·2)	118 (62)	80 (51)
Fever	21,819 (59·6)	20,662 (60·1)	626 (51·4)	97 (51)	71 (45)
Skin and/or mucosal lesions[1]	19,121 (52·2)	18,071 (52·6)	703 (57·7)	110 (58)	74 (47)
Genital rash	16,993 (46·4)	16,201 (47·1)	329 (27·0)	14 (7)	11 (7)
Headache	12,016 (32·8)	11,195 (32·6)	492 (40·4)	47 (25)	44 (28)
Any lymphadenopathy	11,196 (30·6)	10,919 (31·8)	247 (20·3)	15 (8)	9 (6)
Muscle ache	9,937 (27·1)	9,283 (27·0)	366 (30·0)	28 (15)	27 (17)
Local lymphadenopathy	7,794 (21·3)	7,594 (22·1)	175 (14·4)	14 (7)	8 (5)
Fatigue	7,438 (20·3)	6,918 (20·1)	172 (14·1)	8 (4)	6 (4)
General lymphadenopathy	7,408 (20·2)	7,260 (21·1)	135 (11·1)	5 (3)	3 (2)
Sore throat	5,018 (13·7)	4,626 (13·5)	192 (15·8)	17 (9)	16 (10)
Chills	3,199 (8·7)	2,773 (8·1)	113 (9·3)	9 (5)	7 (4)
Oral rash	3,109 (8·5)	2,606 (7·6)	64 (5·3)	4 (2)	8 (5)
Asymptomatic	851 (2·3)	715 (2·1)	73 (6·0)	23 (12)	32 (20)

1. Excluding oral and genital lesions

Table S3: Frequency of reported symptoms among mpox cases by age and sex. Cell values are shown as the number of cases (n) reporting a given symptom, alongside percentage of all cases presenting with that symptom. Case denominators for all cases are shown for each

demographic group in each column header. Some symptoms, most notably chills, fatigue, genital rash, and muscle ache varied substantially by age and sex.

Supplementary figures

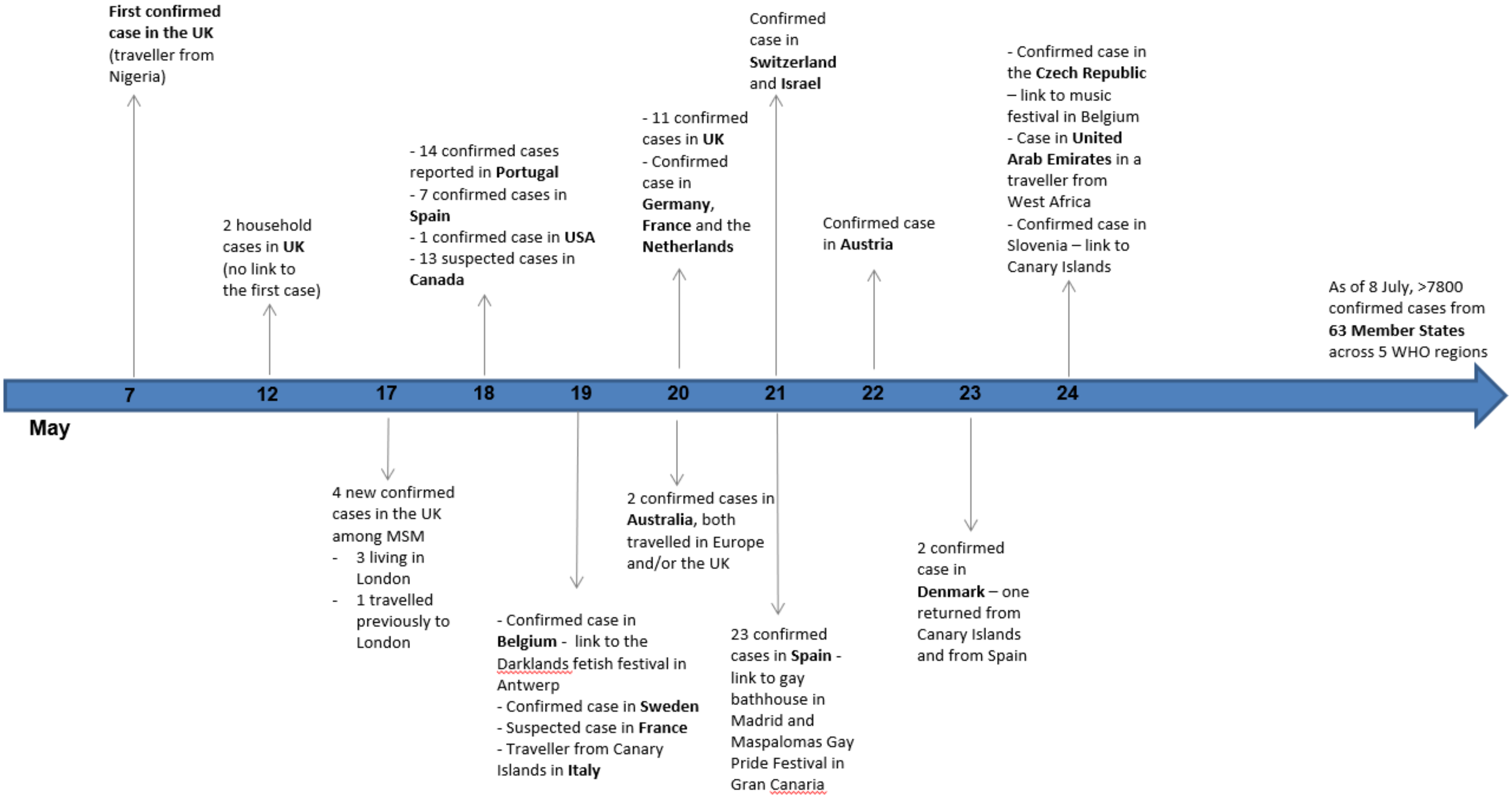

Figure S1: Timeline of the early stages of the global mpox outbreak as reported to WHO, 2022

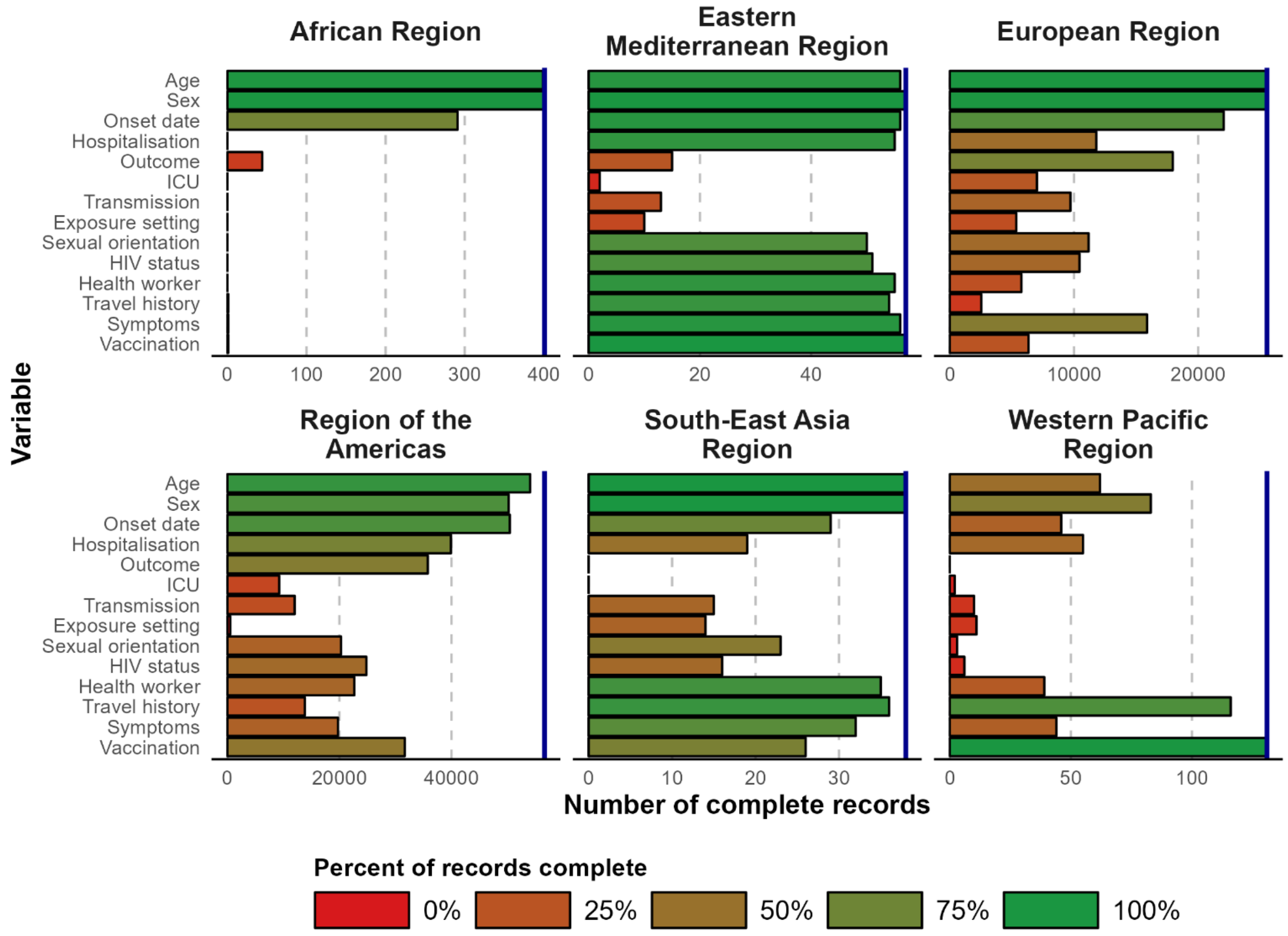

Figure S2: Completeness of case reporting form reporting variables, by WHO region. Where unknown was provided as a response to any variable, it was considered missing. The blue line represents the total number of case reporting forms (CRF) reported for a given WHO region. Bar shading represents percentage of forms with that variable completed. Note that figure should be interpreted knowing total numbers of reported cases varied substantially by region. Within region, completeness was variable by country.

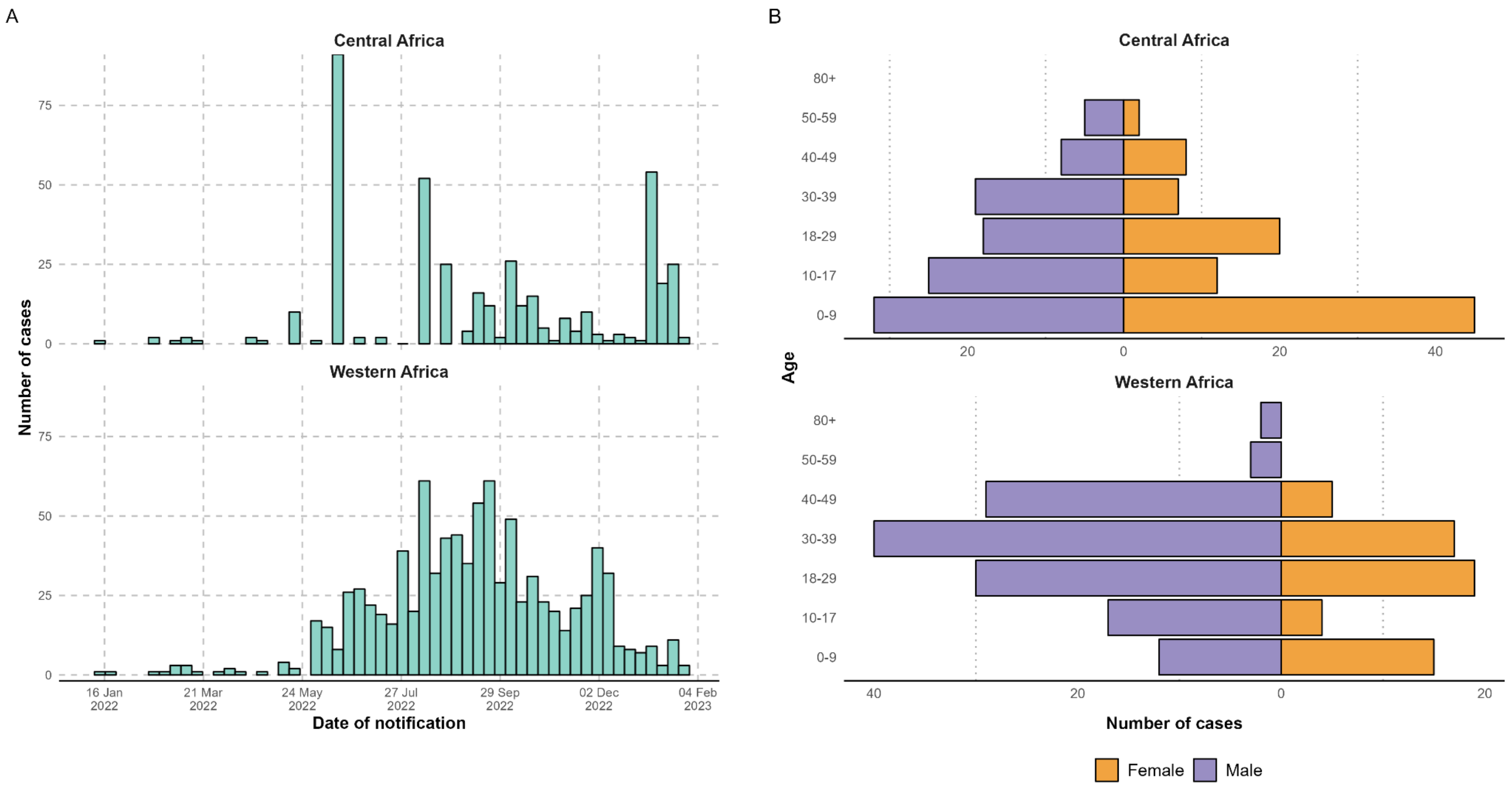

Figure S3: A) Epidemic curves of confirmed mpox cases and age and sex distributions for Central and West Africa. B) Age and sex distributions derived from Western Africa (Benin, Ghana, Liberia, and Nigeria) and Central Africa (Cameroon, Central African Republic [CAR], Congo, and Democratic Republic of the Congo[DRC]).

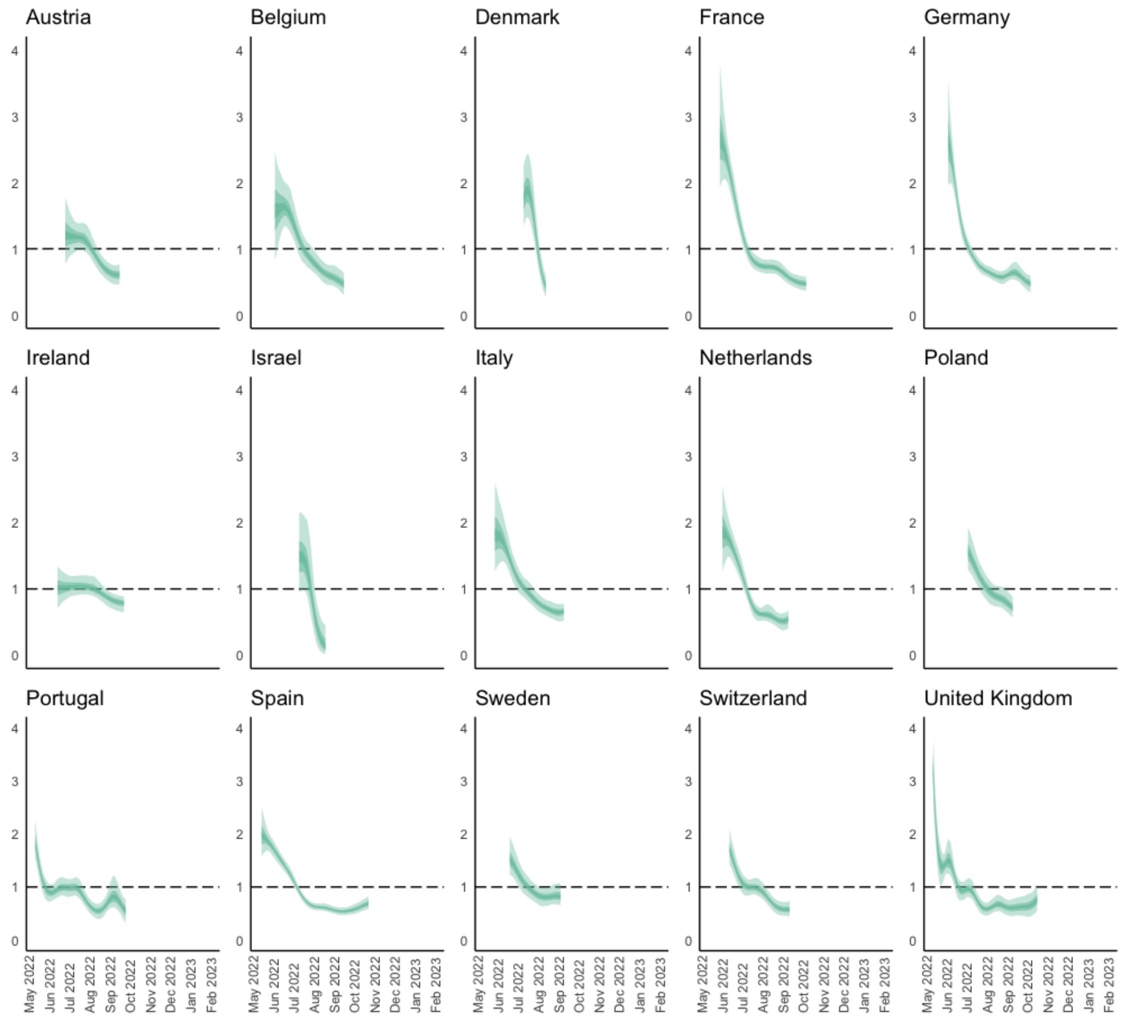

Figure S4a: Estimates of R_{eff} for mpox transmission for European region countries where modelling inclusion criteria have been met. Green corresponds with estimates based on data that is considered largely complete. 20%, 50%, and 90% credible intervals correspond with the darkest, lighter, and lightest shaded areas respectively. Estimates are only shown for weeks in which ten or more cases were observed by a country to preserve quality of estimates.

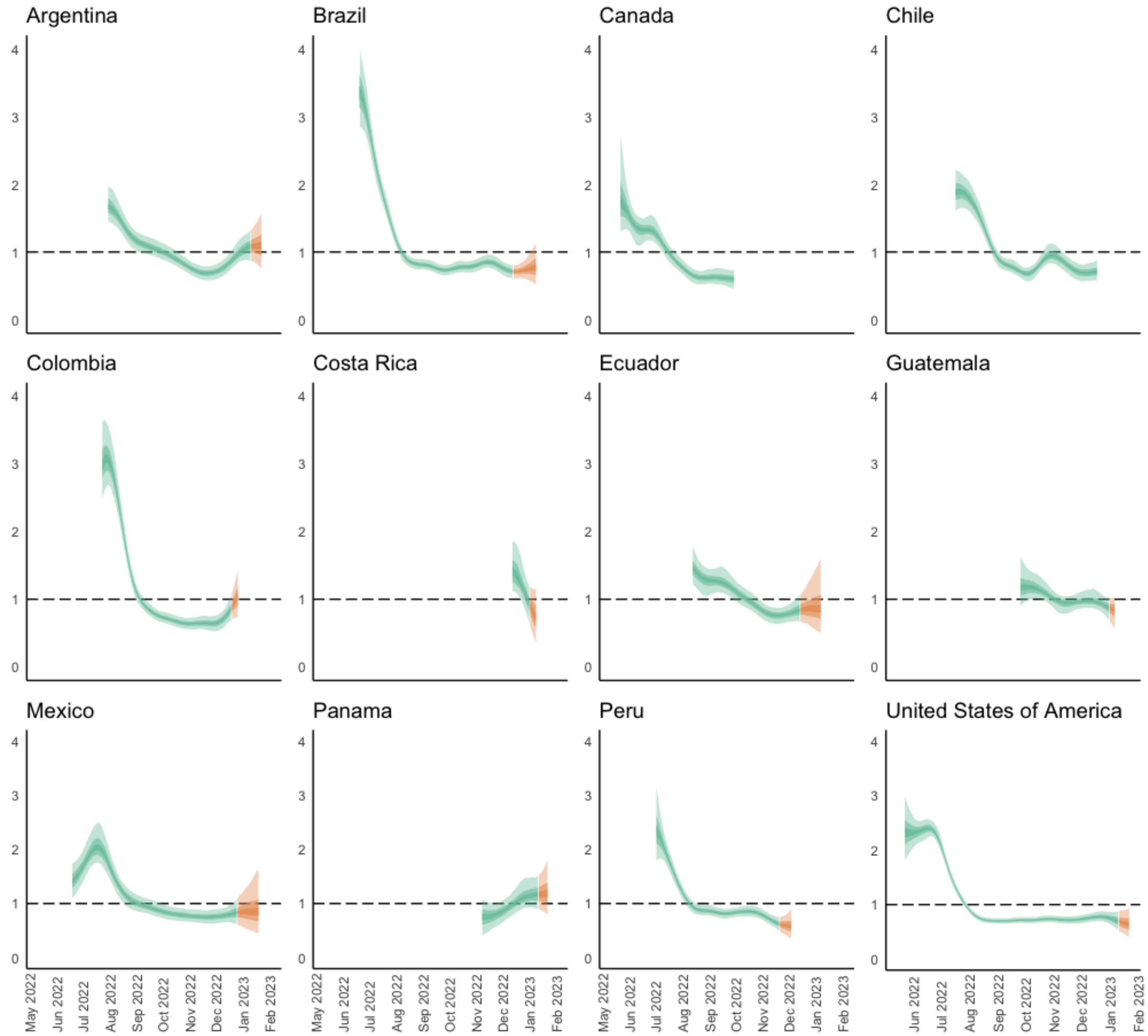

Figure S4b: Estimates of R_{eff} for mpox transmission for Americas region countries where modelling inclusion criteria have been met. Green corresponds with estimates based on data that is considered largely complete and orange corresponds with estimates based on partial data. 20%, 50%, and 90% credible intervals correspond with the darkest, lighter, and lightest shaded areas respectively. Estimates are only shown for weeks in which ten or more cases were observed by a country to preserve quality of estimates.
